# Behavioral Activation and Falls Prevention for Homebound Older Adults With Depression

**DOI:** 10.1093/geroni/igaf122.1993

**Published:** 2025-12-31

**Authors:** Namkee Choi, Kelly Vences, Angelina Gutierrez, Brian Fons

**Affiliations:** The University of Texas at Austin, Austin, Texas, United States; The University of Texas School of Social Work, Austin, Texas, United States; The University of Texas at Austin, Austin, Texas, United States; The University of Texas at Austin, Austin, Texas, United States

## Abstract

This presentation focuses on the implementation and preliminary outcomes of an ongoing four-arm randomized controlled trial of short-term behavioral activation (BA) and multi-component falls prevention (FP) for homebound older adults, age 50 and older, with depression (24-item Hamilton Rating Scale for Depression [HAMD] >15). Both BA and FP are delivered by trained lay counselors via video-conferencing. iPads with a video-conferencing platform and an exercise app, “Keep on Keep up” are loaned to the participants. The study arms are BA only, FP only, combined BA and FP (TBF), and telephone support call which serves as attention control. BA is designed to increase engagement in value-based activities, and FP uses the CDC’s STEADI tool kits and the exercise app. The expected enrollment goal is 320 by March 1, 2026. We have used a variety of recruitment

**Methods:**

referrals from Meals on Wheels case workers, distribution of the study fliers with Meals on Wheels, and presentations at local senior apartment complexes. Barriers to recruitment and retention included mental illness self-stigma and related denial of depression, lack of motivation, chronic pain, and physical health crises. Each study participant goes through baseline assessment, weekly sessions over nine weeks, and three follow-up assessments over nine months. The average age of the participants is 68 years; 70% female and 30% male; 55% Black or Hispanic and 45% non-Hispanic White; and the baseline HAMD scores of 23. We will present 12- and 24-week outcomes. Qualitative feedback from participants in BA, FP, and TBF have been highly positive.

